# Traditional Chinese Medicine is an Alternative Therapeutic Option for Treatment of *Pseudomonas aeruginosa* Infections

**DOI:** 10.3389/fphar.2021.737252

**Published:** 2021-08-27

**Authors:** Zheng Pang, Qingjun Zhu

**Affiliations:** ^1^ Innovative Institute of Chinese Medicine and Pharmacy, Shandong University of Traditional Chinese Medicine, Jinan, China; ^2^ Key Laboratory of Traditional Chinese Medicine Classical Theory, Ministry of Education, Shandong University of Traditional Chinese Medicine, Jinan, China; ^3^ Shandong Provincial Key Laboratory of Traditional Chinese Medicine for Basic Research, Shandong University of Traditional Chinese Medicine, Jinan, China

**Keywords:** traditional Chinese medicine, *Pseudomonas aeruginosa*, quorum sensing, biofilm, bactericidal effects, immunomodulation

## Abstract

*Pseudomonas aeruginosa* is an opportunistic pathogen causing life-threatening infections in cystic fibrosis patients and immunocompromised individuals, and it is a leading cause of nosocomial infections associated with significant morbidity and mortality. Treatment of *P. aeruginosa* infections is challenging due to the antibiotic resistance to most of the conventional antibiotics. Development of alternative therapeutic options is urgently demanded for the patients who have antibiotic-resistant infections. Traditional Chinese medicine (TCM) has a clinical history of thousands of years for prevention and treatment of infectious diseases in China, taking advantages of improving clinical outcomes, producing less side effects, inhibiting pathogen, and modulating host immunity. Recent research has revealed a variety of natural products derived from TCM showing significant antimicrobial effects on antibiotic-resistant strains of *P. aeruginosa* alone or combined with antibiotics *in vitro* or in animal models, suggesting that TCM is a promising complementary and alternative therapeutic approach for treatment of chronic *P. aeruginosa* infections. This review summarizes the recent findings attempting to dissect the mechanisms of TCM combating *P. aeruginosa* infections and highlights the molecular targets of TCM on *P. aeruginosa* and host.

## Introduction

*Pseudomonas aeruginosa* is a Gram-negative, rod-shaped, aerobic bacterium that is commonly found in soil and aqueous environments, and is capable of surviving in harsh conditions with minimum nutrition requirement owing to its numerous metabolic pathways and regulatory genes (Moradali et al., 2017). *Pseudomonas aeruginosa* is also an opportunistic pathogen that can reside on human skin without causing harm in most healthy individuals but causes life-threatening acute infections in immunocompromised individuals and chronic infections in cystic fibrosis (CF) patients (Sadikot et al., 2005). The nosocomial infections caused by *P. aeruginosa* is a major problem in intensive care units with significant mortality and morbidity, which contributes 10–20% of total nosocomial infection cases (Bodey et al., 1983). According to the data from the National Nosocomial Infections Surveillance (NNIS) System, *P. aeruginosa* is the second most common cause of nosocomial pneumonia (17%), the third most common cause of urinary tract infection (7%), and the fourth most common cause of surgical site infection (8%)(Richards et al., 1999; National Nosocomial Infections Surveillance, 2004). Importantly, this bacterial pathogen is able to counter many of the currently available antibiotics such as aminoglycosides, quinolones and β-lactams through intrinsic and acquired resistance mechanisms (Pang et al., 2019). The intrinsic mechanisms include low permeability of outer membrane, expression of efflux pumps and production of antibiotic-inactivating enzymes. The acquired resistance mechanisms are achieved by mutational changes or acquisition of resistance genes *via* horizontal gene transfer, which make the empirical antibiotic treatment become increasingly more difficult and costly (Pang et al., 2019). In particular, the bacterial efflux pumps expelling a broad spectrum of antibiotics from the cell can greatly contribute to multidrug resistance, and they can be classified into five families: resistance-nodulation-division (RND) family, major facilitator superfamily (MFS), ATP-binding cassette (ABC) superfamily, small multidrug resistance (SMR) family, and multidrug and toxic compound extrusion (MATE) family (Sun J. et al., 2014). Furthermore, the RND efflux pumps play a key role in antibiotic resistance in *P. aeruginosa*, which confer the resistance to a variety of antibiotics including aminoglycosides, quinolones, β-lactams, macrolides, novobiocin, chloramphenicol, tetracyclines, trimethoprim, sulfonamides and lincomycin (Masuda et al., 2000; Terzi et al., 2014). Although the newly developed antibiotics with novel mechanisms of action have shown increased effectiveness in bacterial killing, *P. aeruginosa* can rapidly evolve resistance and escape from antibiotic targeting through chromosomal mutations, especially in presence of high concentration of antibiotics (Sanz-Garcia et al., 2018; Botelho et al., 2019). *Pseudomonas aeruginosa* is recognized as one of the six ESKAPE pathogens, including *Enterococcus faecium* (E), *Staphylococcus aureus* (S), *Klebsiella pneumoniae* (K), *Acinetobacter baumannii* (A), *Pseudomonas aeruginosa* (P) and *Enterobacter Species* (E), known for their antimicrobial resistance, and it has been listed by World Health Organization (WHO) as one of the priority bacterial pathogens for research and development of new antibiotics (Tacconelli et al., 2018).

The exploration of complementary and alternative therapeutic strategies against *P. aeruginosa* infections has gained a lot of research attention over the past decade. Many alternative therapeutic approaches including suppression of quorum sensing, inhibition of bacterial lectins, iron chelation, phage therapy, vaccine strategy and nanoparticle application have been assessed and showed antimicrobial activity against multidrug-resistant (MDR) *P. aeruginosa in vitro* or in animal models (Pang et al., 2019). However, most of them are far from application due to many concerns regarding the cost, side effects and safety. Development of new therapeutic options is still urgently demanded. Traditional Chinese medicine (TCM) has a history over three thousand years, which is a comprehensive health care system comprising of herbal medicine, acupuncture, moxibustion, cupping, massage, and physical exercise (Tang et al., 2008). TCM has a unique therapeutic approach to prevent or treat many diseases such as cancer (Xiang et al., 2019), infectious diseases (Ma et al., 2019), cardiovascular diseases (Hao et al., 2017), Alzheimer’s disease (Howes et al., 2017) and diabetes (Tian et al., 2019) by maintaining or restoring the yin-yang balance. For instance, Liao et al. conducted a retrospective cohort study on 111,564 patients with newly diagnosed lung cancer and found that the patients who received TCM treatment had a 32% lower mortality rate compared to the non-TCM users (Liao et al., 2017). Cardiovascular diseases are the leading cause of death worldwide (Mc Namara et al., 2019). Clinical evidence indicated that the TCM Tiankuijiangya tablet displayed a significant anti-hypertensive effect, which significantly reduced the systolic and diastolic blood pressure in the patients with hypertension by 17.64 and 11.85 mm Hg, respectively, compared to placebo controls (Hao et al., 2017). Alzheimer’s disease is a progressive neurodegenerative disease that is irreversible form of dementia, causing impaired memory and cognitive functions (Cass, 2017). Le Bars et al. demonstrated that 27–37% of the patients with Alzheimer’s disease treated with EGb 761, an extract of *Ginkgo biloba L.* leaves (Ginkgoaceae), manifested improved cognitive performance and social functioning compared with placebo controls for 6 months to 1 year (Le Bars et al., 1997). More importantly, TCM has a curative effect on infectious diseases with a long history of rich experience, taking advantages of improvement of clinical outcomes, symptom relief, less side effects, pathogen inhibition, and promotion of host immunity during drug-resistant infections (Yu et al., 2017; Ma et al., 2019). Nowadays, a number of important drugs developed from TCM had significant achievements in public health by controlling many serious infectious diseases. For instance, a Chinese scientist Tu Youyou won the 2015 Nobel Prize for the discovery of artemisinin, a drug extracted from *Artemisia annua* L. (Compositae) leaves for malaria treatment (Tu, 2016). Furthermore, TCM has been clinically applied for treatment of acute or chronic pulmonary infections in China (Yu et al., 2014; Ma et al., 2019; Ren et al., 2020). During COVID-19 pandemic, greater than 85% of SARS-CoV-2-infected patients in China had received TCM treatment, and clinical evidence indicated that the TCM herbal formulas *Qing Fei Pai Du Tang* and *Lian Hua Qing Wen* capsule could significantly alleviate the symptoms, reduce the inflammation, and promote the recovery of COVID-19 patients, and both TCM formulas displayed an effective rate over 90% for COVID-19 treatment (Yang Y. et al., 2020; Du et al., 2020; Li et al., 2020; Ren et al., 2020; Wu et al., 2020).

**FIGURE 1:**
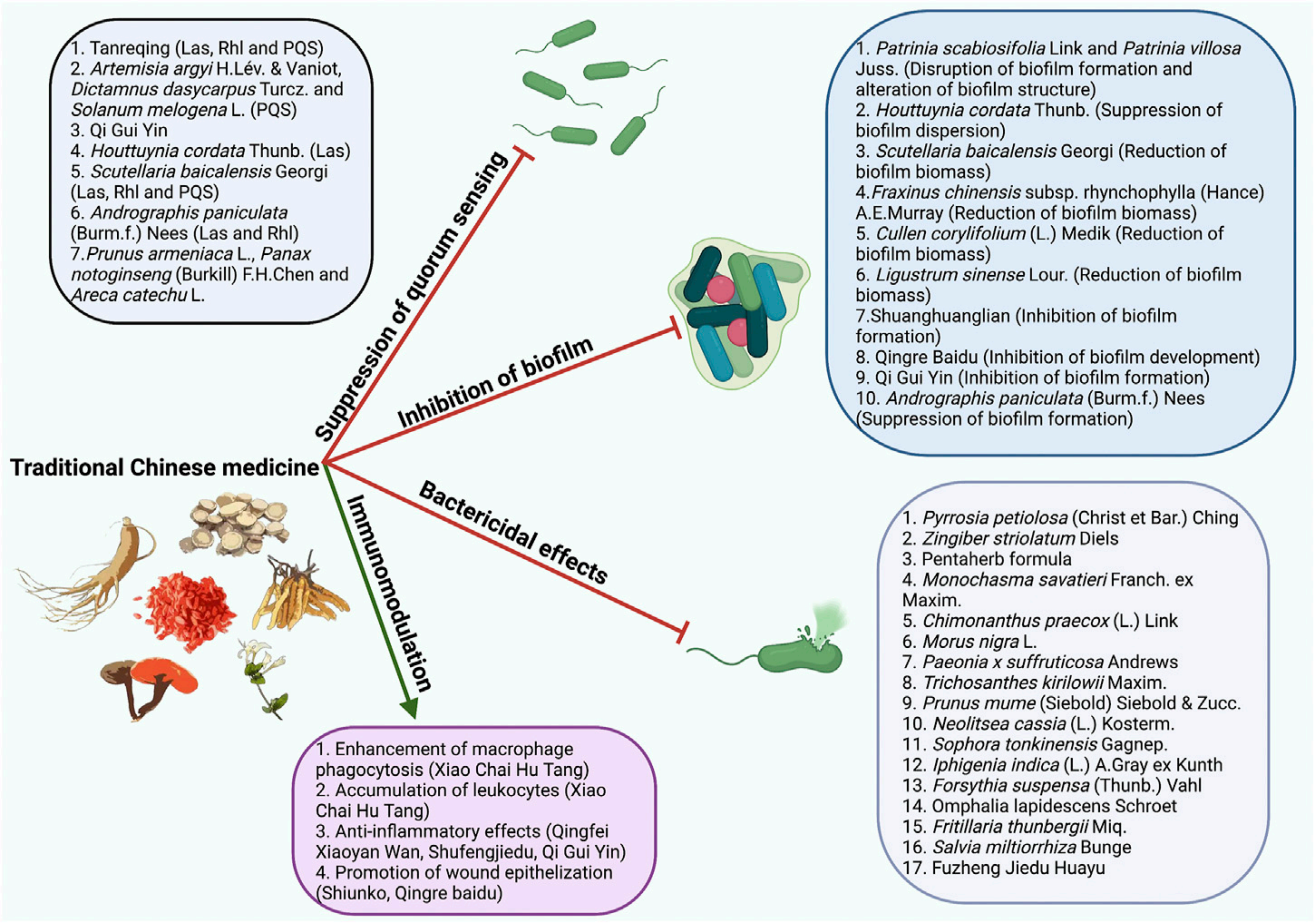
The mechanisms of TCM combating *P. aeruginosa* infections. TCM controls *P. aeruginosa* infections through suppression of QS, inhibition of biofilm, bactericidal effects, and modulation of host immune responses.

TCM has been reported to be capable of effectively controlling *P. aeruginosa* infections through suppression of quorum sensing (QS)(Wei et al., 2020), inhibition of biofilm (Fu et al., 2017), bactericidal effects (Liu et al., 2013), and modulation of host immunity (Hou et al., 2016) (Figure 1). The present review aimed to summarize and discuss the recent findings on the mechanisms of TCM underlying the prevention and treatment of *P. aeruginosa* infections. The scientific names and corresponding common names of the TCM presented in this review were summarized in Table 1.

**TABLE 1:** Summaries of the scientific and common names of TCM ingredients.

TCM scientific names	TCM common names
*Lonicera japonica* Thunb.	Japanese honeysuckle, golden-and-silver honeysuckle
*Scutellaria baicalensis* Georgi	Baikal skullcap, Chinese skullcap
*Forsythia suspensa* (Thunb.) Vahl	Weeping forsythia, golden-bell
Pulvis Fellis Ursi	Bear bile
Cornu Saigae Tataricae	Antelope’s Horn
*Artemisia argyi* H.Lév. & Vaniot	Silvery wormwood or Chinese mugwort
*Dictamnus dasycarpus* Turcz.	Dittany bark
*Solanum melongena* L.	Eggplant
*Astragalus mongholicus* Bunge	Mongolian milkvetch
*Angelica sinensis* (Oliv.) Diels	Chinese angelica, Dang Gui
*Artemisia annua* L.	Sweet wormwood, sweet annie, annual wormwood
*Reynoutria japonica* Houtt.	Japanese knotweed
*Houttuynia cordata* Thunb.	Fish mint, fish leaf, rainbow plant, chameleon plant, heart leaf, fish wort, Chinese lizard tail
*Andrographis paniculata* (Burm.f.) Nees	Creat, green chiretta, king of bitters
*Prunus armeniaca* L.	Apricot
*Panax notoginseng* (Burkill) F.H.Chen	Chinese ginseng, notoginseng
*Areca catechu* L.	Betelnut palm
*Patrinia scabiosifolia* Link	Patrinia, eastern valerian
*Patrinia villosa* Juss.	Patrinia
*Fraxinus chinensis* subsp*.* rhynchophylla (Hance) A.E.Murray	Chinese ash
*Cullen corylifolium* (L.) Medik	Babchi, Bakuchi
*Viola mandshurica* W.Becker	Manchurian Violet, sumire
*Paeonia lactiflora* pall.	Chinese peony
*Salvia miltiorrhiza* Bunge	Red sage, Chinese sage, Tanshen, Danshen
*Gleditsia sinensis* Lam.	Chinese honey locust
*Glycyrrhiza glabra* L.	Liquorice, licorice
*Pyrrosia petiolosa* (Christ et Bar.) Ching	Tongue fern, Japanese felt fern
*Zingiber striolatum* Diels	Yang-he
*Mentha canadensis* L.	American wild mint
*Paeonia* × *suffruticosa* Andrews	Moutan peony
*Atractylodes lancea* (Thunb.) DC.	Southern tsangshu
*Phellodendron chinense* C.K.Schneid	Chinese corktree
*Monochasma savatieri* Franch. ex Maxim.	Not applicable
*Chimonanthus praecox* (L.) Link	Wintersweet
*Morus nigra* L.	Black mulberry
*Trichosanthes kirilowii* Maxim.	Chinese snake gourd
*Prunus mume* (Siebold) Siebold & Zucc*.*	Chinese plum
*Neolitsea cassia* (L.) Kosterm	Grey bollywood
*Sophora tonkinensis* Gagnep.	Vietnamese sophora
*Iphigenia indica* (L.) A.Gray ex Kunth	Not applicable
Omphalia lapidescens Schroet	Omphalia, Leiwan, Thunder Ball
*Fritillaria thunbergii* Miq.	Zhejiang fritillary
*Echinops latifolius* Tausch	Not applicable
*Panax quinquefolius* L.	American ginseng
*Coix lacryma-jobi* var. *ma-yuen* (Rom.Caill.) Stapf	Not applicable
*Ephedra sinica* Stapf	Chinese ephedra
Gypsum Fibrosum	Gypsum
Pheretima	Earthworm
*Arctium lappa* L.	Greater burdock
*Descurainia sophia* (L.) Webb ex Prantl	Flixweed
Bovis Calculus Artifactus	Artificial ox bezoar
*Pinellia ternata* (Thunb.) Makino	Crow-dipper
*Ziziphus jujuba* Mill.	Chinese jujube
*Bupleurum chinense* DC.	Chinese thoroughwax
*Panax ginseng* C.A.Mey.	Asian ginseng, Chinese ginseng, Korean ginseng
*Glycyrrhiza glabra* L.	Liquorice, licorice
*Zingiber officinale* Roscoe	Garden ginger

### Suppression of Quorum Sensing

QS is a bacterial cell-cell communication mechanism that regulates bacterial gene expression in a population density-dependent manner (Miller and Bassler, 2001). *Pseudomonas aeruginosa* utilizes four QS systems, LasI/LasR, RhlI/RhlR, PQS/MvfR and IQS, to activate expression of many virulence factors, including pyocyanin, pyoverdine, elastases, alkaline protease, lectins, rhamnolipids and exotoxin A, which promote bacterial invasion and impair host immune response (Whiteley et al., 1999; Rutherford and Bassler, 2012; Lee et al., 2013). Specifically, *P. aeruginosa* pyocyanin is redox-active phenazine that is not only involved in maintaining bacterial fitness and facilitating biofilm formation but also interferes host cellular functions, such as electron transport, cellular respiration, ciliary function and inflammatory response (Rada and Leto, 2013; Jayaseelan et al., 2014). Pyoverdine is the major siderophore of *P. aeruginosa* that obtains extracellular iron from environment and host proteins, important for bacterial growth and virulence (Meyer, 2000). The elastases produced by *P. aeruginosa* are capable of damaging host tissues and degrading plasma proteins, including immunoglobulins, coagulation factors and complement proteins (Wretlind and Pavlovskis, 1983). Alkaline protease not only damages host tissues but also facilitates bacterial immune evasion through proteolytic cleavage of monomeric flagellin, thus impairing the Toll-like receptor five signaling (Bardoel et al., 2012). Lectins are adhesion molecules located on bacterial outer membrane, which recognize and bind to host glycoconjugates, allowing the attachment of bacteria to host cell surface (Imberty and Varrot, 2008). Rhamnolipids are a class of glycolipid biosurfactants mainly produced by *P. aeruginosa*, and they have multiple functions involved in modification of surface properties, modulation of bacterial swarming motility, disruption of biofilm and alteration of epithelial tight junction (Zulianello et al., 2006; Alhede et al., 2014). Exotoxin A is a highly toxic virulence factor of *P. aeruginosa* that is secreted into the extracellular environment and inhibits host protein synthesis by catalyzing ADP-ribosylation of elongation factor 2 (Michalska and Wolf, 2015).

**FIGURE 2:**
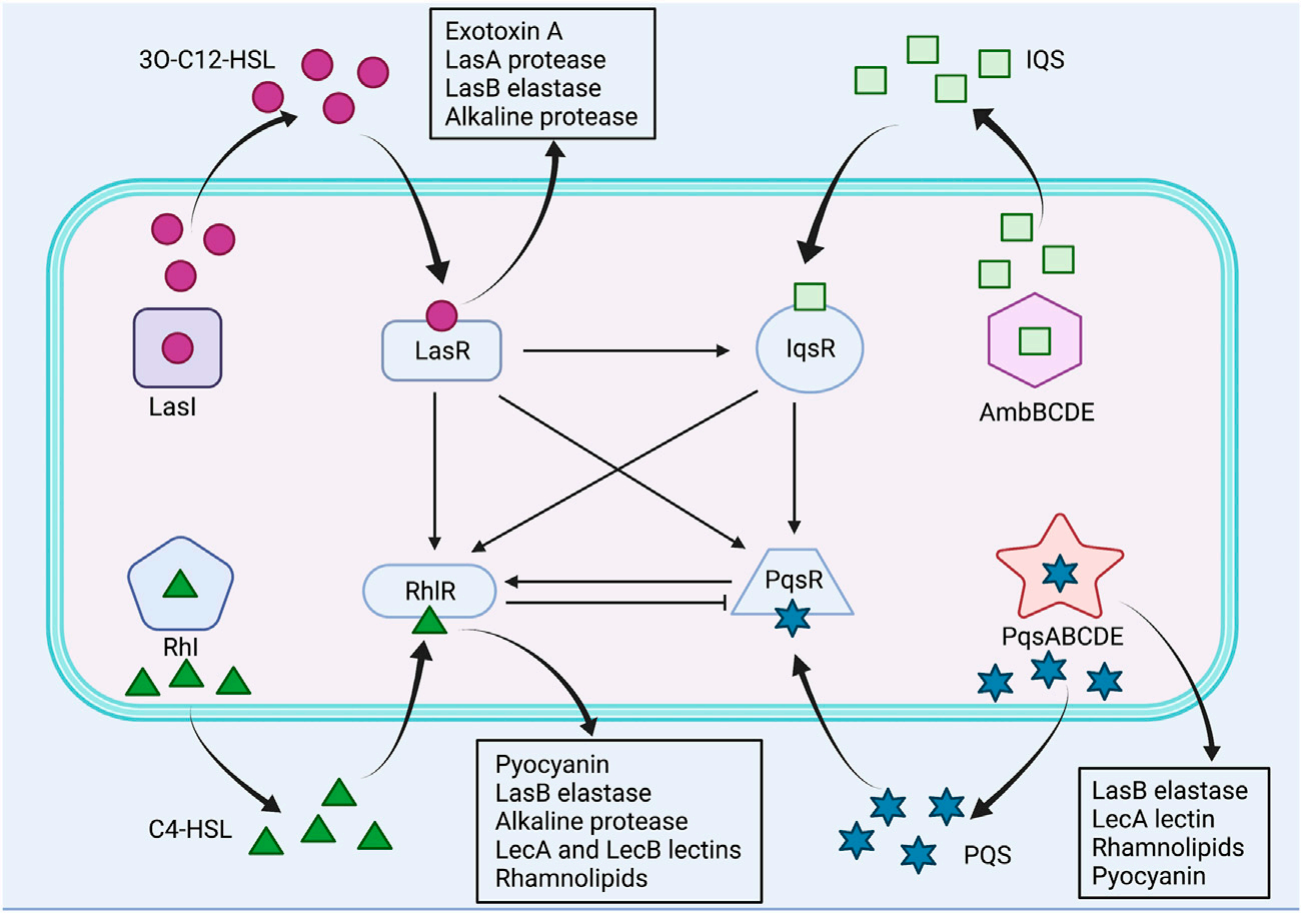
The QS systems of *P. aeruginosa* and their interactions. *Pseudomonas aeruginosa* utilizes four QS systems, including LasI/LasR, RhlI/RhlR, PQS/MvfR and IQS systems. Las system positively regulates Rhl, PQS and IQS systems. Rhl system negatively regulates PQS system, whereas PQS system positively regulates Rhl system. IQS system positively regulates Rhl and PQS systems. Positive control is represented by arrows, and negative control is represented by blunted arrows.

The LasI/LasR and RhlI/RhlR are two canonical LuxI/LuxR QS circuits in *P. aeruginosa*, which catalyze the synthesis of N-acyl homoserine lactone (AHL) autoinducers, N-(3-oxododecanoyl)-L-homoserine lactone (3O-C12-HSL) and N-butanoyl-L-homoserine lactone (C4-HSL), respectively (Waters and Bassler, 2005; Lee and Zhang, 2015; Moradali et al., 2017). Subsequently, the signal molecules 3O-C12-HSL and C4-HSL bind to their respective cognate transcriptional regulators LasR and RhlR, and activate expression of QS-controlled virulence genes (Lee and Zhang, 2015). The Las system regulates the expression of virulence factors, including exotoxin A, LasA protease, LasB elastase and alkaline protease, whereas the Rhl system regulates the expression of the pyocyanin, LasB elastase, alkaline protease, LecA and LecB lectins, and rhamnolipids (Pearson et al., 1997; Glessner et al., 1999; Winzer et al., 2000; Kariminik et al., 2017). Moreover, Las system has shown to positively regulate Rhl system (Lee and Zhang, 2015). By contrast, the third QS signal, *Pseudomonas* quinolone signal (PQS), structurally identified as 2-heptyl-3-hydroxy-4-quinolone, is synthesized through multiple operons, including pqsABCDE, phnAB and pqsH, and it binds to the receptor PqsR, also known as MvfR, to activate production of PQS itself and virulence factors, such as LasB elastase, rhamnolipids, lecA lectin and pyocyanin (Lee and Zhang, 2015; Lin et al., 2018). Furthermore, the PQS synthesis was found to be negatively regulated by Rhl system and positively regulated by Las system, whereas PQS positively regulated Rhl system (McKnight et al., 2000; Wade et al., 2005). The fourth QS signal, integrated quorum sensing system (IQS), structurally characterized to be 2-(2-hydroxyphenyl)-thiazole-4-carbaldehyde, is synthesized *via* a gene cluster ambBCDE, and it is responsible for integrating bacterial stress response with the QS network (Lee and Zhang, 2015). In addition, IQS was identified to be tightly controlled by Las system under rich culture conditions but is activated by phosphate limitation stress, subsequently upregulating Rhl and PQS systems (Lee et al., 2013). The *P. aeruginosa* QS systems were illustrated in Figure 2.

Many Chinese herbal medicines have been reported to possess anti-QS activities (Table 2) (Koh and Tham, 2011). Tanreqing injection has been widely used as an herbal formula for the treatment of viral pneumonia in China, which consists of the extracts from the flower bud of *Lonicera japonica* Thunb. (Caprifoliaceae; *Lonicerae Japonicae Flos*), the root of *Scutellaria baicalensis* Georgi (Lamiaceae; *Scutellariae Radix*), the fruit of *Forsythia suspensa* (Thunb.) Vahl (Oleaceae; *Forsythiae Fructus*), Pulvis Fellis Ursi (Bear bile), and Cornu Saigae Tataricae (Antelope’s Horn) (Liu et al., 2020). Moreover, the side effects of Tanreqing injection include anaphylaxis, drug eruption, nausea, vomiting and arrhythmia (Liu et al., 2020). A recent study by Yang et al. showed that the five components of Tanreqing played the major role in inhibiting the three *P. aeruginosa* systems, Las, Rhl and PQS, through repression of the upstream two-component systems GacS/GacA and PprA/PprB, leading to decreased expression of QS-regulated virulence genes in *P. aeruginosa*. Furthermore, the authors demonstrated a *Caenorhabditis elegans*-*P. aeruginosa* slow-killing assay and found that the survival rate of the *C. elegans* fed with Tanreqing-treated *P. aeruginosa* was significantly increased by 30% compared to those fed with untreated *P. aeruginosa*, suggesting that Tanreqing reduced the virulence of *P. aeruginosa* and protected *C. elegans* from killing (Yang W. et al., 2020). However, the original concentration of Tanreqing injection was not provided in this study. Wei et al. introduced a dry distillation oil prepared from a mixture of the leaf of *Artemisia argyi* H.Lév. & Vaniot (Compositae; *Artemisiae argyi Folium*), the root bark of *Dictamnus dasycarpus* Turcz. (Rutaceae, *Dictamni radicis cortex*) and the root of *Solanum melongena* L. (Solanaceae) with a mixing ratio of 1 : 1 : 2 (1 part of *Artemisiae argyi Folium*, 1 part of *Dictamni radicis cortex*, and 2 parts of the root of *Solanum melongena* L.) by heating in a flask with condenser tube at 350°C, and they found the oil was able to inhibit *P. aeruginosa* PQS system by interrupting the binding of the PQS receptor PqsR to its corresponding promoter pqsA (Wei et al., 2020). No side effects of the three TCM herbs have been reported. Qi Gui Yin is a mixture of Chinese herbal medicines, comprising of the root of *Astragalus mongholicus* Bunge (Leguminosae, *Astragali Radix*), the root of *Angelica sinensis* (Oliv.) Diels (Apiaceae, Angelicae sinensis Radix), the flower bud of *L. japonica* Thunb. (Caprifoliaceae; Lonicerae Japonicae Flos), *Artemisia annua* L. (Compositae), and the root and rhizome of *Reynoutria japonica* Houtt. (Polygonaceae; *Polygoni Cuspidati Rhizoma et Radix*) at a ratio of 12:3: 3:2:2, and it has been found to effectively eliminate antibiotic-resistant *P. aeruginosa* strains (Kong et al., 2015; Ding et al., 2021a). The safety of Qi Gui Yin decoction has been tested on rats, and no toxic and side effects were observed at a dose of 14.3 g for every kg of body weight for 13 weeks (Ding et al., 2021b). Ding et al. analyzed the protein expression profiles of the *P. aeruginosa* strains treated or not treated with Qi Gui Yin decoction, and found the QS-associated proteins, PhzA, PhzB, PhzM and MetQ1, were downregulated in Qi Gui Yin-treated strains. Further study demonstrated that the serum from Qi Gui Yin-treated rats could effectively reduce the resistance of *P. aeruginosa* to imipenem (Ding et al., 2021a). However, the mechanisms underlying the Qi Gui Yin-mediated QS inhibition were not specified in this study, and the metabolites in the serum of Qi Gui Yin-treated rats need to be further characterized. Sodium houttuyfonate is a bioactive compound derived from *Houttuynia cordata* Thunb. (Saururaceae), a well-known TCM botanical drug in East Asia. A study by Wu et al. reported that Sodium houttuyfonate was able to inhibit the expression of *P. aeruginosa* LasI and LasR, leading to impaired production of QS-regulated virulence factors, including pyocyanin and LasA (Wu et al., 2014). It is noteworthy that *H. cordata* Thunb. may cause allergic reactions in some people (Shingnaisui et al., 2018). Baicalin is an active compound purified from *S. baicalensis* Georgi, a famous TCM known for its antioxidant, anti-inflammatory and anticoagulant properties (Kong et al., 2014; Lee et al., 2015). Furthermore, baicalin has been found to inhibit the Las, Rhl and PQS systems of *P. aeruginosa* by downregulating the expression of QS regulatory genes, including *lasI*, *lasR*, *rhlI*, *rhlR*, *pqsR* and *pqsA* (Luo et al., 2017). However, *S. baicalensis* Georgi may cause stomach discomfort, diarrhoea and drug eruption in individual patients (Zhao et al., 2019). *Andrographis paniculata* (Burm.f.) Nees (Acanthaceae) is a medicinal plant widely used in many Asian countries, including China, India and Thailand, and it has been extensively applied in treatment of upper respiratory infections (Jiang et al., 2021)*,* and it has no obvious toxic and side effects on human and animals (Jayakumar et al., 2013). Zhang et al. identified that the andrographolide compounds, andrographolide, 14-deoxyandrographolide, 14-deoxy-12-hydroxyandrographolide and neoandrographolide, from *A. paniculata* (Burm.f.) Nees suppressed the gene expression of LasR in the clinical isolates of *P. aeruginosa* PA22 and PA247 (Tan Lim et al., 2021). Additionally, Banerjee et al. indicated that the chloroform extract of *A. paniculata* (Burm.f.) Nees effectively decreased the expression of *lasI, lasR*, *rhlI* and *rhlR* by 61, 75, 41, and 44% in *P. aeruginosa* PAO1, respectively (Banerjee et al., 2017). QS has been found to regulate bacterial swarming motility, which is a flagella-driven movement of bacterial cells on a moist surface (Daniels et al., 2004). Koh et al. used swarming motility as an indicator of QS activity to screen the QS inhibitory effects of TCM plants, and they found that acetone and water (1:1 ratio) extracts from the seed of *Prunus armeniaca* L. (Rosaceae), the flower and root of *Panax notoginseng* (Burkill) F.H.Chen (Araliaceae), and the seed of *Areca catechu* L. (Arecaceae) impaired the swarming motility of *P. aeruginosa* PAO1 (Koh and Tham, 2011). However, the inhibitory mechanisms of these TCM botanical drugs were not addressed. Additionally, the side effects of *P. armeniaca* L. remain unknown, whereas *P. notoginseng* (Burkill) F.H.Chen has shown toxic effects on liver and kidney (Wang et al., 2016), and *A. catechu* L. may cause diarrhea, stomach discomfort and nausea (Samappito et al., 2012).

**TABLE 2:** Summaries of the TCM for suppression of *P. aeruginosa* QS.

TCM	Suppression of QS	Mechanisms	References
Tanreqing	Las, Rhl and PQS	Repression of QS two-component systems GacS/GacA and PprA/PprB	Yang et al. (2020a)
Dry distillation oil from the leaf of *Artemisia argyi* H.Lév. & Vaniot*.*, the root bark of *Dictamnus dasycarpus* Turcz. and the root of *Solanum melongena* L.	PQS	Interruption of the binding of PqsR to pqsA promoter	Wei et al. (2020)
Qi Gui Yin	Not specified	Downregulation of expression of PhzA, PhzB, PhzM and MetQ1	Ding et al. (2021a)
Sodium houttuyfonate from *Houttuynia cordata* Thunb.	Las	Inhibition of LasI and LasR expression	Wu et al. (2014)
Baicalin from *Scutellaria baicalensis* Georgi	Las, Rhl and PQS	Downregulation of transcription of *lasI, lasR, rhlI, rhlR, pqsR* and *pqsA*	Luo et al. (2017)
Andrographolide compounds from *Andrographis paniculata* (Burm.f.) Nees	Las and Rhl	Inhibition of *lasI, lasR*, *rhlI* and *rhlR* expression	Banerjee et al. (2017); Tan Lim et al. (2021)
Seed of *Prunus armeniaca* L., flower and root of *Panax notoginseng* (Burkill) F.H.Chen, and seed of *Areca catechu* L.	Not specified	Impairment of *P. aeruginosa* swarming motility	Koh and Tham, (2011)

### Inhibition of Biofilm

**FIGURE 3:**
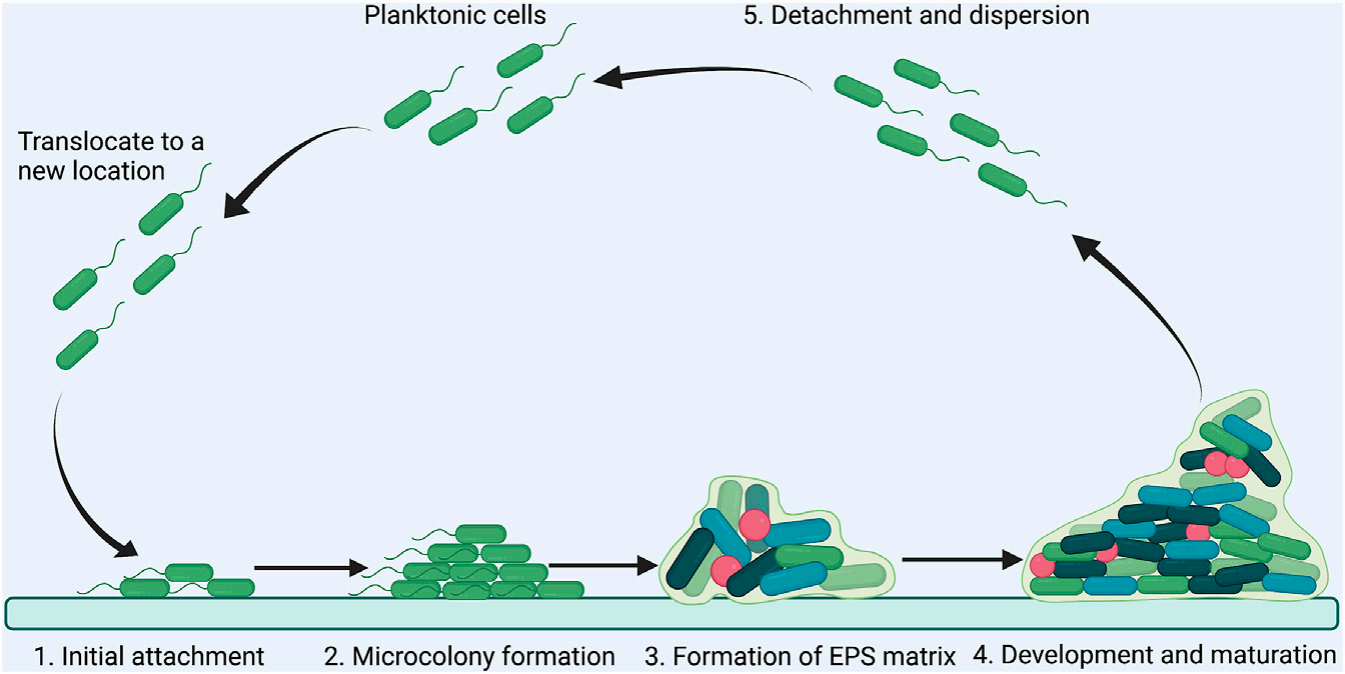
Schematic illustration of the steps involved in *P. aeruginosa* biofilm formation. The formation of *P. aeruginosa* biofilm has five steps: **(A)** initial attachment of planktonic bacteria to a surface; **(B)** microcolony formation; **(C)** formation of EPS matrix; **(D)** development and maturation; **(E)** detachment and dispersion.

A biofilm is an aggregate of microorganisms in which cells adhere to each other on a static surface, and the adherent cells are embedded within a self-produced matrix of extracellular polymeric substances (EPS), consisting of polysaccharides, proteins, DNA and lipids (Donlan, 2002). Generally, biofilm formation can be divided into five steps: 1) initial attachment of planktonic bacteria to a surface; 2) microcolony formation; 3) formation of matrix by producing EPS; 4) maturation of biofilm into a three-dimensional structure; 5) detachment and dispersion of the biofilm (Figure 3)(Jamal et al., 2018; O'Toole et al., 2000). Bacteria in biofilm are more resistant to antimicrobial agents than planktonic cells because of the poor antibiotic penetration, reduced metabolic and growth rates, induction of adaptive stress responses, and formation of persister cells in biofilm microenvironment (Stewart, 2002; Hoiby et al., 2010). Importantly, approximate 80% of chronic infections are associated with biofilm formation (Jamal et al., 2018). Disruption of biofilm is critical for controlling persistent bacterial infections.

*Pseudomonas aeruginosa* is a major cause of chronic respiratory infections in CF patients, leading to declined pulmonary function and ultimate mortality (Lyczak et al., 2002). Formation of *P. aeruginosa* biofilm is regulated by QS systems, two-component regulatory systems, exopolysaccharides and bis-(3′–5′)-cyclic dimeric guanosine monophosphate (c-di-GMP)(Rasamiravaka et al., 2015). Two-component regulatory systems consist of a sensor kinase and a response regulator, which are essential for signaling transduction in bacteria in response to environment stimuli (Mikkelsen et al., 2011). The two-component regulatory systems GacS/GacA and RetS/LadS are responsible for regulation of *P. aeruginosa* biofilm formation (Rasamiravaka et al., 2015). The exopolysaccharides produced by *P. aeruginosa* include alginate, Pel and Psl, which contribute to biofilm development and stabilize biofilm architecture (Ghafoor et al., 2011). The c-di-GMP is a nucleotide second messenger that regulates plentiful cellular processes in bacteria, and high levels of c-di-GMP repress bacterial motility and induce production of exopolysaccharides, which promote *P. aeruginosa* biofilm formation (Ha and O'Toole, 2015).

TCM botanical drugs have manifested inhibitory effects on *P. aeruginosa* biofilm (Table 3)(Fu et al., 2017; Wang T. et al., 2019; Xu Z. et al., 2019). Herba patriniae, also known as Bai Jiang Cao, is a TCM for heat-clearing and detoxication, consisting of two species of Caprifoliaceae family, *Patrinia scabiosifolia* Link (Caprifoliaceae) and *Patrinia villosa* Juss. (Caprifoliaceae) (Gong et al., 2021). Fu et al. reported that the water extract of Herba patriniae was able to disrupt the biofilm formation and alter the biofilm structure of *P. aeruginosa* PAO1 through inhibition of exopolysaccharide production and biofilm-associated genes including *algU*, *pslM*, *pelA*, *algA*, and *bdlA* (Fu et al., 2017). However, the authors did not specify the species of Herba patriniae in this study. Additionally, the mild side effects including temporary leukopenia, dizziness and nausea could be found in the patients treated with inappropriate dose of Herba Patriniae (Gong et al., 2021). As mentioned earlier, sodium houttuyfonate from *H. cordata* Thunb. could inhibit *P. aeruginosa* QS systems (Wu et al., 2014). A study by Wang et al. identified that sodium houttuyfonate was able to penetrate *P. aeruginosa* biofilm and suppress biofilm dispersion by inhibiting the expression of the key biofilm regulator BdlA (Wang T. et al., 2019). Moreover, Wu et al. set up a rat model of *P. aeruginosa* biofilm infections in airway, and they found that sodium houttuyfonate destructed biofilm formation and the sodium houttuyfonate-treated rat displayed reduced symptoms and pulmonary inflammation (Wu et al., 2016). Zeng et al. examined the effects of several TCM chemical compounds on *P. aeruginosa* biofilm and found that the Baicalin and Baicalein from *S. baicalensis* Georgi, the Esculetin and Esculin from *Fraxinus chinensis* subsp*.* rhynchophylla (Hance) A.E.Murray (Oleaceae), and the Psoralen from *Cullen corylifolium* (L.) Medik (Leguminosae) could significantly reduce the mass of *P. aeruginosa* biofilm (Zeng et al., 2008). The mechanisms involved in the reduction of biofilm biomass by the three active compounds need to be further explored. Furthermore, the side effects of *F. chinensis* subsp*.* rhynchophylla (Hance) A.E.Murray and *C. corylifolium* (L.) Medik have not yet been clinically reported. *Ligustrum sinense* Lour. (Oleaceae) is an evergreen shrub used in TCM for their anti-oxidative, anti-tumor, and diuretic properties (Ouyang et al., 2003; Yang et al., 2013), and the side effects of this TCM are not clear. A previous study revealed that the water-soluble extract of *L. sinense* Lour. (4 mg/ml) could strongly decrease the biomass of *P. aeruginosa* biofilm, and 1.35 mg/ml of this extract displayed a synergistic inhibitory effect with gentamycin sulphate (2 mg/ml) on the growth of *P. aeruginosa* PAO1 (Yang et al., 2013). Shuanghuanglian is an antiviral and antibacterial TCM formula comprised of the flower bud of *L. japonica* Thunb., the root of *S. baicalensis* Georgi, and the fruit of *F. suspensa* (Thunb.) Vahl (Yan et al., 2013), and Shuanghuanglian injection has been reported to induce skin allergic reactions (Wang et al., 2010). Xu et al. demonstrated that the Lonicerin, a flavonoid from the Shuanghuanglian component *L. japonica* Thunb., inhibited the alginate secretion of *P. aeruginosa* by sequestering alginate secretion protein AlgE, thus leading to reduced *P. aeruginosa* biofilm formation (Xu Z. et al., 2019). Qingre Baidu mixture is a TCM formula composed of *A. sinensis* (Oliv.) Diels, *Viola mandshurica* W.Becker (Violaceae), the flower bud of *L. japonica* Thunb., *Paeonia lactiflora* pall. (Paeoniaceae), *Salvia miltiorrhiza* Bunge (Lamiaceae; *Salviae miltiorrhizae radix et rhizome*), *F. suspensa* (Thunb.) Vahl, the root of *A. mongholicus* Bunge, *Gleditsia sinensis* Lam. (Leguminosae) and *Glycyrrhiza glabra* L. (Leguminosae)(Zhang et al., 2020), and the adverse reactions of this TCM formula are unclear. Shan et al. applied Qingre Baidu mixture to a rat model of refractory wound through oral administration with a dose of 40 mg for every kg of body weight, and discovered that Qingre Baidu mixture could inhibit the biofilm formation of *P. aeruginosa* through downregulation of a self-inducer molecule AI2, important for regulation of biofilm development in *P. aeruginosa* (Shan et al., 2019). In addition, the QS inhibitory TCM Qi Gui Yin has exhibited downregulation of the expression of biofilm-related gene *ArcA* and *IscU* in *P. aeruginosa* (Ding et al., 2021a). The andrographolide compounds from *A. paniculata* (Burm.f.) Nees mentioned earlier could inhibit the biofilm formation of *P. aeruginosa* PA22 by 69–84% and PA247 by 23–56% at a concentration of 5 mg/ml (Tan Lim et al., 2021), and the chloroform extract of *A. paniculata* (Burm.f.) Nees inhibited the biofilm of *P. aeruginosa* PAO1 by 75.2% at a concentration of 1.25 mg/ml (Banerjee et al., 2017)*.*

**TABLE 3:** Summaries of the TCM for inhibition of *P. aeruginosa* biofilm.

TCM	Effects on biofilm	Mechanisms	References
*Patrinia scabiosifolia* Link and *Patrinia villosa* Juss.	Disruption of biofilm formation and alteration of biofilm structure	Inhibition of exopolysaccharide production, and the expression of *algU*, *pslM*, *pelA*, *algA*, and *bdlA*	Fu et al. (2017)
Sodium houttuyfonate from *Houttuynia cordata* Thunb.	Suppression of biofilm formation and dispersion	Inhibition of BdlA expression	Wu et al. (2016); Wang et al. (2019b)
Baicalin and Baicalein from *Scutellaria baicalensis* Georgi	Reduction of biofilm biomass	Not specified	Zeng et al. (2008)
Esculetin and Esculin from *Fraxinus chinensis* subsp*.* rhynchophylla (Hance) A.E.Murray	Reduction of biofilm biomass	Not specified	Zeng et al. (2008)
Psoralen from *Psoralea corylifolia* L.	Reduction of biofilm biomass	Not specified	Zeng et al. (2008)
*Ligustrum sinense* Lour.	Reduction of biofilm biomass	Not specified	Yang et al. (2013)
Lonicerin from Shuanghuanglian	Inhibition of biofilm formation	Inhibition of alginate secretion by sequestering AlgE	Xu H. et al. (2019)
Qingre Baidu mixture	Inhibition of biofilm development	Downregulation of AI2	Shan et al. (2019)
Qi Gui Yin	Inhibition of biofilm formation	Downregulate of expression of *ArcA* and *IscU*	Ding et al. (2021a)
Andrographolide compounds from *Andrographis paniculata* (Burm.f.) Nees	Suppression of biofilm formation	Not specified	Banerjee et al. (2017); Tan Lim et al. (2021)

### Bactericidal Effects

A variety of TCMs have shown bactericidal effects against respiratory pathogens by directly killing or inhibiting their growth (Ooi et al., 2006; Liu et al., 2007; Yamada et al., 2016), and some of them could enhance the *in vitro* antibacterial effects of antibiotics in treatment of MDR *P. aeruginosa* infections (Liu et al., 2007; Xu H. et al., 2019), possibly due to the enhanced stability of antibiotics by the reductive components of TCM (Gim et al., 2009; Chen et al., 2013; Matkowski et al., 2013; Sun W. et al., 2014; Chen et al., 2020), and the inhibitory effects of TCM on bacterial efflux pumps (Huang et al., 2015; Wang et al., 2018; Wang J. et al., 2019), suggesting that TCM could be a good alternative or complement for synthetic antibiotics. The minimum inhibitory concentration (MIC) and minimum bactericidal concentration (MBC) values of TCM against *P. aeruginosa* were summarized in Table 4.

**TABLE 4:** Summaries of the bactericidal effects on TCM on *P. aeruginosa*.

TCM	MIC	MBC	References
*Pyrrosia petiolosa* (Christ et Bar.) Ching	5 mg/ml	5 mg/ml	Cheng et al. (2014)
Rhizomes of *Zingiber striolatum* Diels	3.12 mg/ml	Not specified	Tian et al. (2020)
Pentaherb formula composed of the flower bud of *Lonicera japonica* Thunb., *Mentha canadensis* L., the root bark of *Paeonia* × *suffruticosa* Andrews, *Atractylodes lancea* (Thunb.) DC. and the bark of *Phellodendron chinense* C.K.Schneid	1 mg/ml	125 mg/ml	Hon et al. (2018)
Phenylethanoid glycosides from *Monochasma savatieri* Franch. ex Maxim.	0.5 mg/ml	2 mg/ml	Liu et al. (2013)
Sesquiterpenoids of *Chimonanthus praecox* (L.) Link	207.9–249.1 ug/ml	Not specified	Lou et al. (2018)
Flavonoids from *Morus nigra* L.	Not specified	2 mg/ml	Chen et al. (2017)
Root bark of *Paeonia* × *suffruticosa* Andrews	3–6 mg/L	Not specified	Liu et al. (2007)
Ripe pericarp of *Trichosanthes kirilowii* Maxim.	15–20 mg/L	Not specified	Liu et al. (2007)
Unripe fruit of *Prunus mume* (Siebold) Siebold & Zucc.	1–3 mg/L	Not specified	Liu et al. (2007)
*Neolitsea cassia* (L.) Kosterm.	4–5 mg/L	Not specified	Liu et al. (2007)
Root and rhizome of *Sophora tonkinensis* Gagnep.	25–30 mg/L	Not specified	Liu et al. (2007)
Bulb of *Iphigenia indica* (L.) A.Gray ex Kunth	>30 mg/L	Not specified	Liu et al. (2007)
Fruit of *Forsythia suspensa* (Thunb.) Vahl	>30 mg/L	Not specified	Liu et al. (2007)
Omphalia lapidescens Schroet	>30 mg/L	Not specified	Liu et al. (2007)
Bulb of *Fritillaria thunbergii* Miq.	15–20 mg/L	Not specified	Liu et al. (2007)
Root of *Salvia miltiorrhiza* Bunge	20–25 mg/L	Not specified	Liu et al. (2007)
Fuzheng Jiedu Huayu	200 mg/ml	Not specified	Xu H. et al. (2019)

*Pyrrosia petiolosa* (Christ et Bar.) Ching (Polypodiaceae) is a pteridophyte used as a TCM for the treatment of nephritis and bronchitis without causing adverse reactions (Cheng et al., 2020), and the ethanol extract of *P. petiolosa* (Christ et Bar.) Ching has shown significant inhibitory effects on *P. aeruginosa* ATCC27853 with an MIC and an MBC both at 5 mg/ml (Cheng et al., 2014). *Zingiber striolatum* Diels (Zingiberaceae) is a perennial plant widely distributed in southern China, and it has been used for many medicinal purposes for its antioxidant and antimicrobial properties without causing side effects (Sharifi-Rad et al., 2017). Tian et al. characterized the chemical components of the essential oil extracted from the rhizomes of *Z. striolatum* Diels by hydrodistillation, including β-phellandrene, sabinene, β-pinene, geranyl linalool, terpinen-4-ol, a-pinene and crypton, and determined the MIC of the essential oil against *P. aeruginosa* to be 3.12 mg/ml (Tian et al., 2020). The TCM Pentaherb formula is composed of the flower bud of *L. japonica* Thunb., *Mentha canadensis* L. (Lamiaceae), the root bark of *Paeonia* × *suffruticosa* Andrews (Paeoniaceae), *Atractylodes lancea* (Thunb.) DC. (Compositae) and the bark of *Phellodendron chinense* C.K.Schneid (Rutaceae) with a mixture ratio of 2:1:2:2:2, and it has been shown to effectively treat atopic dermatitis without causing side effects (Hon et al., 2007). Hon et al. determined the MIC and MBC of the water extracts of the Pentaherb formula against *P. aeruginosa* ATCC 27853 to be 1 mg/ml and 125 mg/ml, respectively (Hon et al., 2018). *Monochasma savatieri* Franch. ex Maxim. (Orobanchaceae) is a perennial botanical drug widely used in TCM for treatment of many inflammatory diseases, and no toxic effects of this botanical drug have been documented (Gao H. et al., 2017). Liu et al. found that *P. aeruginosa* ATCC 27853 was sensitive to the phenylethanoid glycosides from *M. savatieri* Franch. ex Maxim., which had an MIC of 0.5 mg/ml and an MBC of 2 mg/ml (Liu et al., 2013). Furthermore, they demonstrated that the survival rates of the mice pretreated with phenylethanoid glycosides at a dose of 180 mg for every kg of body weight were significantly increased by 75% compared to untreated mice following *P. aeruginosa* intraperitoneal infections, and the pre-treatment of this drug could reduce the bacterial burden in the lung tissues of the *P. aeruginosa* lung-infected mice (Liu et al., 2013). *Chimonanthus praecox* (L.) Link (Calycanthaceae), also known as wintersweet, is a deciduous shrub used for treatment of coughs, rheumatic arthritis, throat wounds, dizziness, nausea and fever, and its side effects are not well-characterized (Kitagawa et al., 2016). The sesquiterpenoids isolated from the ethyl acetate extract of *C. praecox* (L.) Link stems and roots have been reported to possess antibacterial effects against *P. aeruginosa* ATCC 10145 with an MIC of 207.9–249.1 ug/ml (Lou et al., 2018). Mulberry is not only a fruit with a lot of nutrients but also a TCM comprising of many antioxidant and anti-inflammatory compounds (Huang et al., 2013), and the MBC of the flavonoids from the fruits of black mulberry *Morus nigra* L. (Moraceae) against *P. aeruginosa* was assessed to be 2 mg/ml (Chen et al., 2017). A research group from Taiwan discovered 10 ethanol extracts of Chinese herbal medicines including the root bark of *Paeonia × suffruticosa* Andrews, the ripe pericarp of *Trichosanthes kirilowii* Maxim. (Cucurbitaceae; *Trichosanthis Pericarpium*), the unripe fruit of *Prunus mume* (Siebold) Siebold & Zucc*.* (Rosaceae), *Neolitsea cassia* (L.). Kosterm. (Lauraceae; *Ramulus Cinnamomi*), the root and rhizome of *Sophora tonkinensis* Gagnep. (Leguminosae; *Sophorae Tonkinensis radix et rhizome*), the bulb of *Iphigenia indica* (L.) A.Gray ex Kunth (Colchicaceae), the fruit of *F. suspensa* (Thunb.) Vahl, Omphalia lapidescens Schroet (Leiwan), the bulb of *Fritillaria thunbergii* Miq. (Liliaceae*; Fritillariae Bulbus*), and the root of *S. miltiorrhiza* Bunge possessed a broad-spectrum of bactericidal activity against antibiotic-resistant *P. aeruginosa* strains. Among these herbal medicines, *N. cassia* (L.) Kosterm. was found to synergize with tetracycline, gentamycin and streptomycin to inhibit *P. aeruginosa* growth (Liu et al., 2007). Of note, *S. tonkinensis* Gagnep. may cause toxic effects including hematotoxicity, neurotoxicity, and immunotoxicity (Zhang et al., 2021), and the bulb of *F. thunbergii* Miq. exhibited toxicity to rats at the doses greater than 1 mg for every kg of body weight (Li et al., 2014). The side effects of other TCM botanical drugs listed above have not been reported. Fuzheng Jiedu Huayu formula consists of eight Chinese botanical drugs, including *S. baicalensis* Georgi, *F. suspensa* (Thunb.) Vahl, *Echinops latifolius* Tausch (Compositae), *T. kirilowii* Maxim., *Panax quinquefolius* L. (Araliaceae), *P. scabiosifolia* Link, and *Coix lacryma-jobi* var. *ma-yuen* (Rom.Caill.) Stapf (Poaceae) with a mixture ratio of 2.5:2.5:2.5:2.5:5:1:5:5 (Xu H. et al., 2019). Furthermore, the Fuzheng Jiedu Huayu formula decoction has been applied to treat elderly patients with pneumonia in clinical trials, and no obvious adverse reactions were manifested (Xu et al., 2018). Xu et al. revealed that the Fuzheng Jiedu Huayu decoction with an MIC of 200 mg/ml against *P. aeruginosa* combined with Imipenem/cilastatin sodium could enhance the *in vitro* bactericidal and bacteriostatic effects of Imipenem/cilastatin sodium on MDR *P. aeruginosa* (Xu H. et al., 2019).

### Immunomodulation

A tightly controlled immune response ensures an effective host defense against microbial infection and maintenance of tissue homeostasis (Liu and Cao, 2016). Excessive immune response causes host tissue damage, septic shock and ultimately death (Bortolotti et al., 2018). On the other hand, deficient immune response results in chronic and persistent bacterial infections (Smith et al., 2009). Immunomodulation is an important function of TCM, which activates or suppresses the immune responses of a variety of immune cells, including macrophages (Jian et al., 2019), dendritic cells (Chen et al., 2006), T cells (Guo et al., 2015), B cells (Kawakita et al., 1987a) and NK cells (Hoffman et al., 2020). The immunomodulatory effects of TCM on host immunity in the context of *P. aeruginosa* infections have been evaluated in past decade (Table 5)(Kong et al., 2015; Hou et al., 2016; Li et al., 2017).

**TABLE 5:** Summaries of TCM-modulated host immune responses during *P. aeruginosa* infections.

TCM	Effects on immune responses	Mechanisms	References
Qingfei Xiaoyan Wan	Reduction of lung inflammation	Suppression of PI3K/AKT and Ras/MAPK pathways, and impairment of production of TNF-α, IL-6) and RANTES	Hou et al. (2016)
Xiao Chai Hu Tang	Accumulation of polymorphonuclear leukocytes and promotion the phagocytic activity of macrophages	Not specified	Kawakita et al. (1987b)
Shufengjiedu capsule	Alleviation of lung inflammation	Inhibition of ERK pathway and NF-κB activation	Li et al. (2017)
Shiunko	Promotion of epithelization in skin wound	Enhancement of fibroblast proliferation and collagen production, and suppression of skin inflammation	Huang et al. (2004); Yan et al. (2015); Imai et al. (2019)
Qi Gui Yin	Increase of B cell response and downregulation of inflammation	Enhancement of antibody reactivity to β-lactamases and reduction of the levels of IL-1β and Th1/Th2 ratio	Kong et al. (2015)
Qingre Baidu mixture	Promotion of angiogenesis and wound healing	Upregulation of the expression of HIF-1α, HIF-2α and VEGF	Shan et al. (2019)

Qingfei Xiaoyan Wan, a TCM pill, consists of *E. sinica* Stapf, Gypsum Fibrosum, Pheretima (earthworm), the ripe fruit of *Arctium lappa* L. (Compositae; *Arctii Fructus*), the seed of *Descurainia sophia* (L.) Webb ex Prantl (Brassicaceae), Bovis Calculus Artifactus (Artificial ox bezoar), the seed of *P. armeniaca* L. and Cornu Saigae Tataricae (Hou et al., 2016), and it has been used in China for treatment of asthma and respiratory tract infections without induing obvious side effects (Zhao et al., 2013). A study by Hou et al. evaluated the effects of Qingfei Xiaoyan Wan suspended in distilled water on a mouse model of *P. aeruginosa*-induced acute pneumonia, and they demonstrated that the oral administration of Qingfei Xiaoyan Wan (18 g for every kg of body weight per day) for 1 week could reduce the *P. aeruginosa* PAK-induced lung inflammation by decreasing the production of cytokines (TNF-α and IL-6) and chemokine (RANTES) in lung tissues, significantly ameliorating lung injury. Importantly, the authors identified arctigenin, cholic acid, chlorogenic acid and sinapic acid to be the anti-inflammatory ingredients of Qingfei Xiaoyan Wan, which suppressed the expression of the regulatory proteins in PI3K/AKT and Ras/MAPK pathways (Hou et al., 2016). Xiao Chai Hu Tang is a 7-ingredient TCM consisting of the tuber of *Pinellia ternata* (Thunb.) Makino (Araceae), the fruit of *Ziziphus jujuba* Mill. (Rhamnaceae), and the root of *S. baicalensis* Georgi, *Bupleurum chinense* DC. (Apiaceae), *Panax ginseng* C.A.Mey. (Araliaceae), *Glycyrrhiza glabra* L. (Leguminosae) and *Zingiber officinale* Roscoe (Zingiberaceae), and it is widely used for treatment of respiratory, hepatic and gastrointestinal diseases (Hsu et al., 2006). However, Xiao Chai Hu Tang has been reported to cause hepatotoxicity due to overdose or long-term use (Gao X. et al., 2017). Kawakita et al. demonstrated that the water extract of Xiao Chai Hu Tang (100 mg for every kg of body weight) was able to protect mice from intraperitoneal infections with *P. aeruginosa* by promoting the accumulation of polymorphonuclear leukocytes and enhancing the phagocytic activity of macrophages (Kawakita et al., 1987b). The underlying mechanisms need to the further explored. Shufengjiedu capsule is an oral Chinese patent medicine that has been extensively utilized for treatment of respiratory infectious diseases and chronic obstructive pulmonary disease without causing serious adverse reactions (Xia et al., 2020). In the context of *P. aeruginosa* pulmonary infections, pretreatment of the power suspension from Shufengjiedu capsule with a dose of 0.09 g for every kg of body weight by intragastric gavage was reported to reduce the *P. aeruginosa* PAK-induced lung inflammation in mice through inhibition of ERK pathway and NF-κB activation, leading to decreased IL-6 production and alleviated lung injury (Li et al., 2017). Furthermore, the active components of Shufengjiedu capsule including verbenalin, phillyrin and emodin were identified to contribute to the regulation of ERK pathway (Li et al., 2017). *Pseudomonas aeruginosa* is one of the common pathogens isolated from chronic wounds, and the infections by *P. aeruginosa* affect wound healing (Serra et al., 2015). Shiunko ointment is a traditional botanic formula used for treatment of wounded skin without causing undesirable side effects in China and Japan (Higaki et al., 1999), and it has been found to accelerate the epithelization of wounded skin infected with *P. aeruginosa* (Huang et al., 2004). The mechanisms underlying the Shiunko-modulated epithelization possibly being through enhancing fibroblast proliferation and collagen production, and suppressing skin inflammation (Yan et al., 2015; Imai et al., 2019). In addition to inhibition of *P. aeruginosa* QS and biofilm formation, Qi Gui Yin decoction (9.9 g for every kg of body weight) was also identified to promote host immune response to *P. aeruginosa* infections by enhancing the antibody reactivity to β-lactamases, including VIM-1, SPM-1, and TEM-1, and manifested anti-inflammatory effects by reducing the levels of IL-1β and Th1/Th2 ratio in a rat model of abdominal infection (Kong et al., 2015). Qingre Baidu mixture was reported to promote angiogenesis and wound healing in a rat model of refractory wounds during *P. aeruginosa* infection by upregulating the expression of HIF-1α, HIF-2α and VEGF (Shan et al., 2019).

## Conclusion

Antibiotic resistance has led to a significant challenge for treatment of *P. aeruginosa* infections in clinical settings. Many alternative therapeutic options have been developed over the past decade and demonstrated promising antimicrobial effects against antibiotic-resistant *P. aeruginosa* infections *in vitro* or in animal models. However, only a few of them has proceeded to clinical trials. By contrast, TCM has been clinically practiced over thousands of years in China with its unique system of theories, diagnostics and therapies for many kinds of diseases. In particular, the Chinese herbal medicines have shown great clinical efficacies in prevention and treatment of chronic infectious diseases. Importantly, TCM was reported to effectively control the infections caused by the clinical isolates of *P. aeruginosa* strains with antibiotic resistance through inhibiting the expression of QS regulatory proteins, disrupting biofilm structure and maturation, directly killing or inhibiting bacterial growth, and modulating host immune responses, including acceleration of the leukocyte accumulation to infection sites, enhancement of macrophage phagocytosis, reduction of host inflammatory response and promotion of wound epithelization. Moreover, some studies indicated that combination of TCM and antibiotics could enhance the effects of antibiotics against *P. aeruginosa* infections, which could be attributed to the antibiotic properties of TCM and the reductive components of TCM stabilizing the antibiotics from degradation (Gim et al., 2009; Chen et al., 2013; Matkowski et al., 2013; Sun W. et al., 2014; Chen et al., 2020). In addition, many TCM active compounds have shown inhibitory effects on the gene expression of bacterial efflux pumps, which could also enhance the antimicrobial effects of antibiotics (Huang et al., 2015; Wang et al., 2018; Wang J. et al., 2019). For instance, baicalin was found to significantly inhibit the mRNA transcription of MsrA efflux pump in *Staphylococcus saprophyticus* (Wang J. et al., 2019). The emodin from *Rheum palmatum* L. (Polygonaceae), the schizandrin from *Schisandra chinensis* (Turcz.) Baill. (Schisandraceae), the berberine from *Berberis vulgaris* L. (Berberidaceae), and baicalin were identified to significantly reduce the expression of the efflux pump gene *hefA* in *Helicobacter pylori* (Huang et al., 2015). However, the role of TCM in regulation of the efflux pumps in *P. aeruginosa* has not yet been elucidated, which could be a direction for future research. Altogether, TCM emerges as a promising complementary and alternative therapeutic approach for treatment of *P. aeruginosa* infections. However, due to the multiple gradients and targets of TCM, the mechanisms involved in the TCM-inhibited *P. aeruginosa* infections are incompletely understood. Future studies need to focus on investigating the effects of a single active component of TCM on *P. aeruginosa* and host, which gives a clearer understanding of the drug-host and drug-microbe interactions, allowing development of the TCM with reduced side effects and improved effectiveness in clinical trials.

## Abbreviations

ABC, ATP-binding cassette; AHL, N-acyl homoserine lactone; C4-HSL, N-butanoyl-L-homoserine lactone; c-di-GMP, bis-(3′-5′)-cyclic dimeric guanosine monophosphate; CF, cystic fibrosis; EPS, extracellular polymeric substances; IQS, integrated quorum sensing system; MATE, multidrug and toxic compound extrusion family; MBC, minimum bactericidal concentration; MDR, multidrug-resistant; MFS, major facilitator superfamily; MIC, minimum inhibitory concentration; 3O-C12-HSL, N-(3-oxododecanoyl)-L-homoserine lactone; NNIS, national nosocomial infections surveillance; PQS, *Pseudomonas* quinolone signal; QS, Quorum sensing; RND, resistance-nodulation-division family; SMR, small multidrug resistance; TCM, traditional chinese medicine; WHO, world health organization.
